# Microparticles and PD1 interplay added a prognostic impact in treatment outcomes of patients with multiple myeloma

**DOI:** 10.1038/s41598-021-96975-4

**Published:** 2021-09-03

**Authors:** Asmaa M. Zahran, Zeinab Albadry M. Zahran, Amal Rayan

**Affiliations:** 1 Clinical Pathology Department, South Egypt Cancer Institute, Assiut University, Assiut, Egypt; 2 Clinical Pathology Department, Faculty of Medicine, Assiut University, Assiut, Egypt; 3 Clinical Oncology Department, Faculty of Medicine, Assiut University, Assiut, Egypt

**Keywords:** Cancer, Immunology, Oncology

## Abstract

Although multiple myeloma (MM) is still considered as an incurable disease by current standards, the development of several combination therapies, and immunotherapy approaches has raised the hope towards transforming MM into an indolent, chronic disease, and possibly achieving a cure. We tried to shed light on the expression of PD1 and different Microparticles (MPs) in MM and their interplay as a mechanism of resistance to standardized treatments, in addition, find their associations with prognostic factors of symptomatic MM. Thirty patients with newly diagnosed and chemotherapy naïve active MM, along with 19 healthy participants of comparable age and sex were recruited, after diagnosis of MM; blood samples were collected from both patients and controls for flow cytometric detection of CD4+, CD8+, CD4+PD1+, and CD8+PD1+T cells, total MPs, CD138+ MPs, and platelet MPs. MM patients had statistically significant higher levels of TMPs, CD138+ MPs compared to their controls, while PMPs exhibited no significant difference between both groups. Statistically significant higher percentages of CD8+, PD1CD8+, PD1CD4+T cells were detected in patients compared to controls, while the latter group had a significantly higher percentage of CD4+T cells than MM patients, patients who did not achieve complete response, had significantly higher percentages of PMPs, CD138+MPs, PD1+CD8+, PD1+CD4+, and CD8+T cells (cutoff values = 61, 10.6, 13.5, 11.3 and 20.1 respectively), (*p*-values = 0.002, 0.003, 0.017, 0.001 and 0.008 respectively). Microparticles and PD1 expressions were associated with proliferative potential and resistance to Bortezomib-based treatments, our results suggested that they played a crucial role in myeloma progression.

## Introduction

Multiple myeloma (MM), a type of malignancy arises from plasma cells in bone marrow, represents about 10% of all hematologic malignancies, standard approach of treatment consists of induction therapy followed by high dose chemotherapy and autologous stem cell transplantation (ASCT) in candidate patients, for non-candidate patients, standard doublet, triplet, or quadruplet, agent-containing induction treatment are applied until progression. Although MM is still considered as an incurable disease by current standards, the development of several combination therapies, and immunotherapy approaches has increased the hope towards transforming MM into an indolent, chronic disease, and possibly achieving a cure in some patients^1^ with a median survival for all patients of about 3–4 years^2^.

In addition to the International and Durie-Salmon staging systems, biological markers, including cytogenetic abnormalities such as presence of translocations including t(4;14), and t(14;16), hypodiploidy, del(17p), and del(13), serum *β*2-microglobulin levels greater than 2.5 mg/L, an elevated plasma cell labeling index, and detection of circulating plasma cells, are predictors of poor prognosis in newly diagnosed MM patients^3,4^.

Microparticles are extracellular vesicles with a size around 0.1 to 1 μm, shed from cell surfaces into intercellular spaces and blood circulation after stimulation^5^, MPs rather, develop as a result of blebbing of plasma membrane^6^, previous studies have reported that MPs provide a “non-genetic” basis for the acquirement, progression and supremacy of detrimental tumor behaviors, including enhanced metastatic potentiality of malignant cells and multidrug resistance (MDR)^7,8^.

CD138, a transmembrane heparin sulfate proteoglycan, is expressed on the surface of mature plasma cells to act as a classical co-receptor for growth factors, angiogenic factors, and chemokines; it is the gold standard marker for detecting MM cells using immunohistochemistry and multiparametric flow cytometry analysis of bone marrow biopsies^9^.

Multiple myeloma is a tumor of elderly with longtime antigen exposure, and subsequent over-expression of PD 1 to indicate myeloma antigen–exposed T cells, viral antigen–specific memory T cells, or immune senescent cells^10^, up-regulation of PD1 on CD8+, CD4+T cells, and NK cells is observed in MM^11^, blocking of PD1 will restore T and NK cell functions. PD-L1, a major ligand of PD1, is upregulated on myeloma cells to become more proliferative, resistant to cell-mediated killing and traditional myeloma drugs^12^. Engagement of PD-1 by its ligands, PD-L1 or PD-L2, results in the activation of phosphatases that deactivate signals emanating from the T-cell receptor^13^, up regulates the expression of basic leucine ATF-like transcription factor (BATF), which in turn impairs T-cell proliferation and cytokine secretion^14^.

We tried to shed light on the expression of PD1 and CD138+ microparticles in MM and their possible interplay as a mechanism of resistance to standardized treatments by proteasome inhibitors and immunomodulatory drugs, in addition, find their associations with other prognostic factors of symptomatic MM.

## Patients and methods

The study was a prospective non randomized controlled one carried out in Assiut university hospital and south Egypt cancer Institute of Assiut University.

### Ethical statement

All methods were carried out in accordance with declarations of Helsinki, the study was approved by ethical committee of faculty of medicine, Assiut university (IRB no: 17300484). Written informed consents were obtained from all participants after explaining our objectives and study procedures.

### Inclusion and exclusion

A total of 30 patients with newly diagnosed chemotherapy naïve active multiple myeloma were recruited over a period of 1 year (from start of January/2019 to end of December/2019), and followed up over a period of 6 months to determine their response to treatments, bisphosphonates and palliative local radiotherapy were allowed, patients with previous history of chemotherapy, targeting therapy, or hormonal therapy for any other malignancies were excluded, also patients with excruciated infections, and non-candidate patients for myeloma therapy were excluded. In addition, 19 healthy participants of comparable age and sex were recruited.

### Methodology

For patients: the diagnosis of symptomatic multiple myeloma was done by bone marrow aspirate ± biopsy for evaluation of plasma cell infiltration, immunophenotyping, flow cytometry, protein electrophoresis, immunofixation electrophoresis, and serum free light chain measurements (done in limited number of cases).

Furthermore, blood samples were collected from both patients and controls for flow cytometric detection of CD4+T cells, CD8+T cells, CD4+PD1+T cells, CD8+PD1+T cells, total Microparticles (TMP), CD138+MPs, and platelet MPs (PMP).

Patients were evaluated for different prognostic factors and hematologic parameters including, CBC, β2 microglobulin, LDH, serum calcium, and blood chemistries (CRAB signs).

Evaluation of bone lysis was done based on whole body magnetic resonance imaging; any focal lesion to be considered of value must be ≥ 5 mm in size.

Patients with performance status ≤ 2 received Bortezomib with dexamethasone or Bortezomib, lenalidomide, and dexamethasone regimens; monthly zoledronic acid was given ± palliative local radiotherapy to alleviate pain. Other supportive measures including recombinant erythropoietin and darbepoitin alfa were used to treat myeloma-associated anemia to maintain Hb around 12 g/dL and below 14 g/dL to avoid thromboembolic complications after measuring their microparticles level, also granulocyte colony-stimulating factor (G-CSF) was used to treat chemotherapy-induced neutropenia.

Patients at high risk of thrombosis, patients with elevated levels of MPs, patients received high dose dexamethasone, also patients under lenalidomide treatment received aspirin to avoid thromboembolic complications.

Complete response (CR) was defined as negative immunofixation on the serum and urine, with disappearance of any soft tissue plasmacytomas and ≤ 5% plasma cells in BM, deviation from this definition of CR was considered non CR.

### Flow cytometric detection of microparticles

Blood samples were collected into a 5 mL tubes containing 3.2% citrate for MPs isolation.

### Microparticles isolation and characterization

. The MPs were isolated within 15 min. after collection. Cells were removed by centrifugation for 20 min at 1550×*g* at 20 °C. Then 250 µL of plasma were centrifuged for 30 min at 18,800×*g* at 20 °C. After centrifugation, the supernatant was removed and the pellet was resuspended in phosphate-buffered saline (PBS) and centrifuged for 30 min at 18,800×*g* at 20 °C. The supernatant was removed again and MPs pellet was suspended in PBS^15^.

Flow cytometric analysis was used to quantify and characterize MPs. Five µL of MPs sample were diluted in 35 µL PBS containing 2.5 mM CaCl_2_. The samples were then incubated for 20 min at room temperature in the dark with 5 µL of fluoroisothiocyanate (FITC)-conjugated annexin V (IQ products, Netherland), 5 μL of phycoerythrin (PE) CD146, and allophycocyanin (APC) conjugated anti CD45 (All from Becton Dickinson Biosciences, USA). 5 µL FITC-conjugated annexin V (IQ products, Netherland), 5 μL of PE conjugated CD138 and peridinium-chlorophyll-protein (Per-CP) conjugated CD41 in separate tubes then PBS/calcium buffer were added and the samples were analyzed on a Fluorescence Activated Cell Sorter (FACS)Caliber flow cytometry with Cell Quest software (Becton Dickinson Biosciences, USA). Anti-human IgG was used as an isotype-matched negative control for each sample. Fifty thousand events were analyzed.

**Figure 1:**
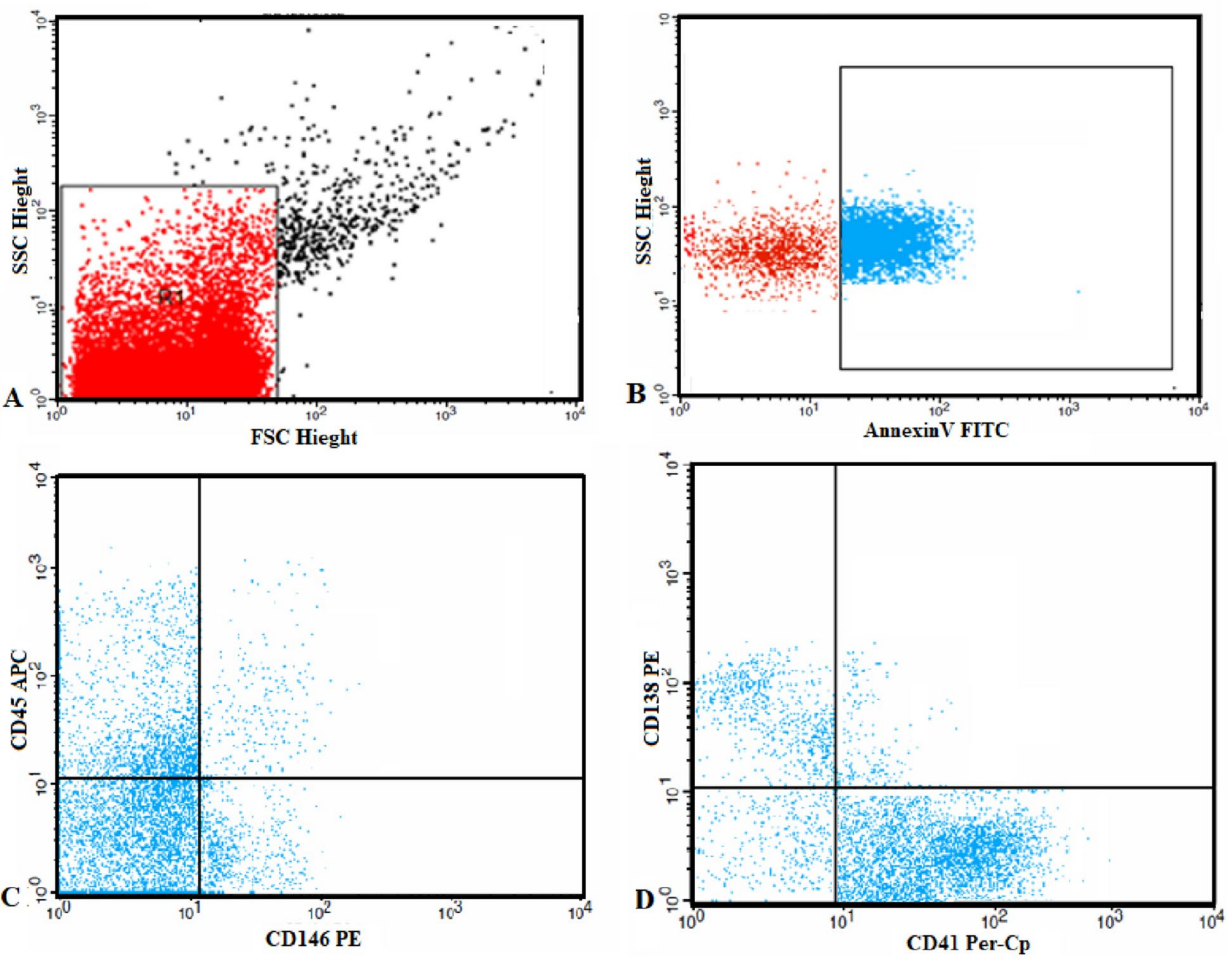
Flow cytometric analysis of circulating microparticles: (**A**) Forward and side scatter histogram was used to define the microparticle**s** (MPs) according to their size (R1). (**B**) Events defined as MPs (R1) were then selected for their annexin V binding. (**C**,**D**) Then annexin V-positive MPs (total MPs) were further examined for expression of cell specific antibodies as CD45, CD146, CD41 and CD138 antibodies to detect endothelial, platelet and myeloma (CD138+MPs).

Total MPs were identified on the basis of their forward scatter compared with that of calibrate reference beads of 1.0 µm to calibrate the size range of microparticles (Latex beads, amine-modified polystyrene, fluorescent red aqueous suspension, 1.0 μm mean particle size, Sigma-Aldrich ChemieGmbh Munich, Germany) and positivity for annexin V. Total MPs were reported as a percentage of the total events. MPs subpopulations were identified by their ability to bind cell-specific monoclonal antibodies. Platelet MPs was detected as CD41+MPs. Myeloma MPs were CD138+MPs. The percentage platelet and myeloma MPs were expressed as percentage of total MPs (Fig. 1).

### Flow cytometric detection of T lymphocyte subsets in peripheral blood

**Figure 2:**
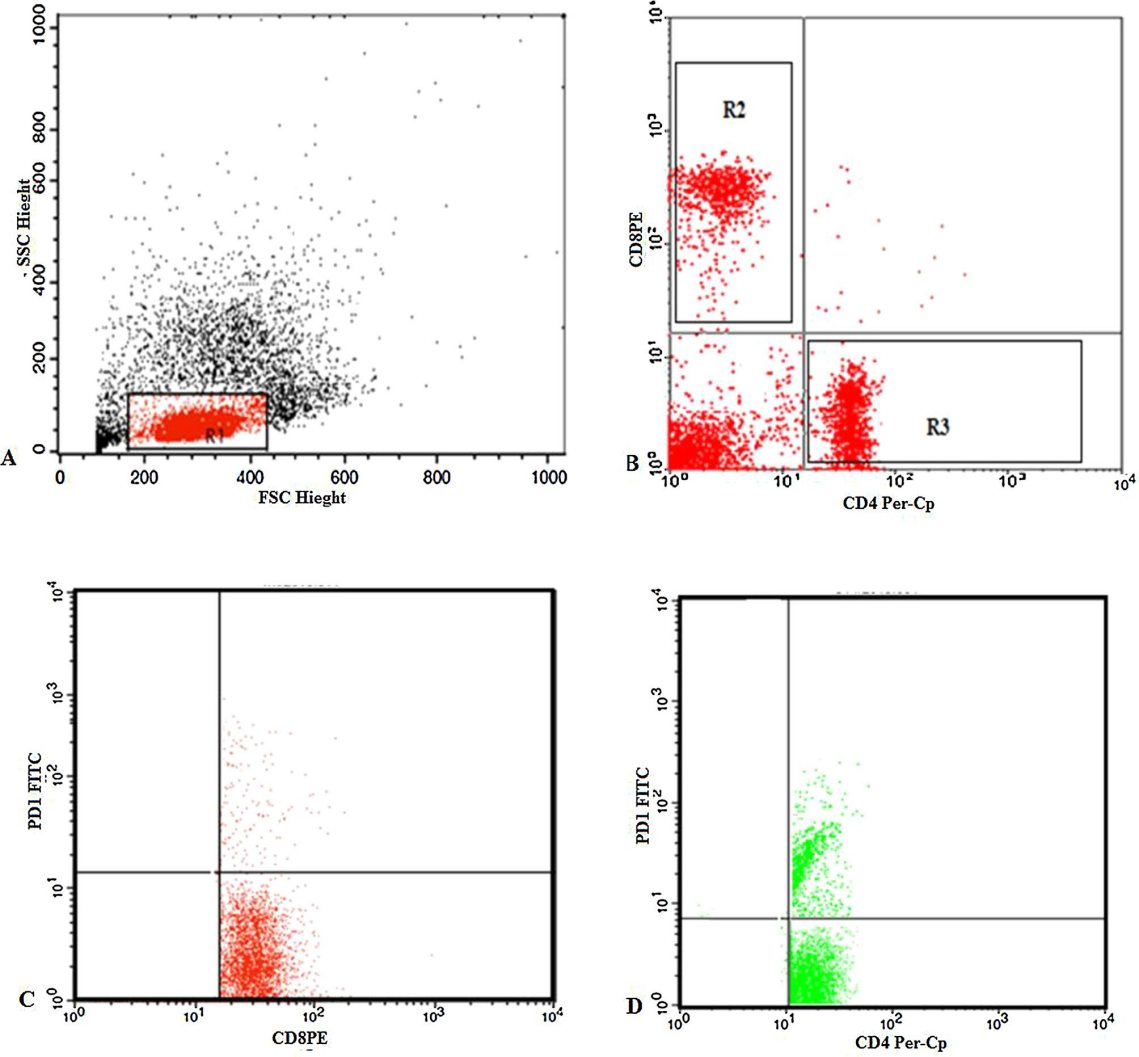
Flow cytometric detection of T lymphocyte subsets and their expression of PD-1 in peripheral blood of multiple myeloma. (**A**) Forward and side scatter histogram was used to define the lymphocytes population. (**B**) The expression of CD4, CD8 on the T lymphocytes was assessed to detected CD4+ (T-helper cells), CD8+ (T-cytotoxic cells). (**C**,**D**) The expression of PD-1 was assesses on CD8+ and CD4+ cells to detect PD1+CD8+T cells and PD1+CD4+T cells.

T lymphocyte subsets in the peripheral blood were assessed by staining 50 µl of blood sample with 5 µl of the following monoclonal antibodies in separate tubes; Fluoroisothiocyanate (FITC)-conjugated-PD-1, phycoerythrin (PE)-conjugated-CD8, peridinium-chlorophyll-protein (Per-CP)-conjugated-CD4. All monoclonal antibodies were purchased from Becton Dickinson (BD) Biosciences, San Jose, CA, USA. After incubation for 20 min at 4 °C in the dark, RBCs lysis was done and washing with phosphate buffer saline (PBS). The cells were resuspended in PBS and analyzed by FACSCalibur flow cytometry with Cell Quest software (BD Biosciences, USA). Human IgG was used as an isotype-matched negative control for each sample. Forward and side scatter histogram was used to define the lymphocyte populations. Then the expression of CD4, CD8 on the T lymphocytes was assessed to detect CD4+ (T-helper cells), CD8+ (T-cytotoxic cells). Then the expression of PD-1 was assesses on CD8+ and CD4+ cells to detect (PD1+CD4+T cells) and (PD1+CD8+T cells) (Fig. 2). The expression was reported as percentages of each cell population.

### Statistics

It was expected that the percentage of MM in Egypt in 2020 according to Amal et al. study^16^ was 0.45% to total number of cancers, so the sample size was calculated based on the equation= $\frac{{z}^{2}pq}{{e}^{2}}$ , where p = 0.0045, q = 0.995, absolute error e = 0.03, and z for 99% confidence interval was.2.56, so the calculated sample size was 32 patients, and we recruited 30 patients for our study.

**Figure 3:**
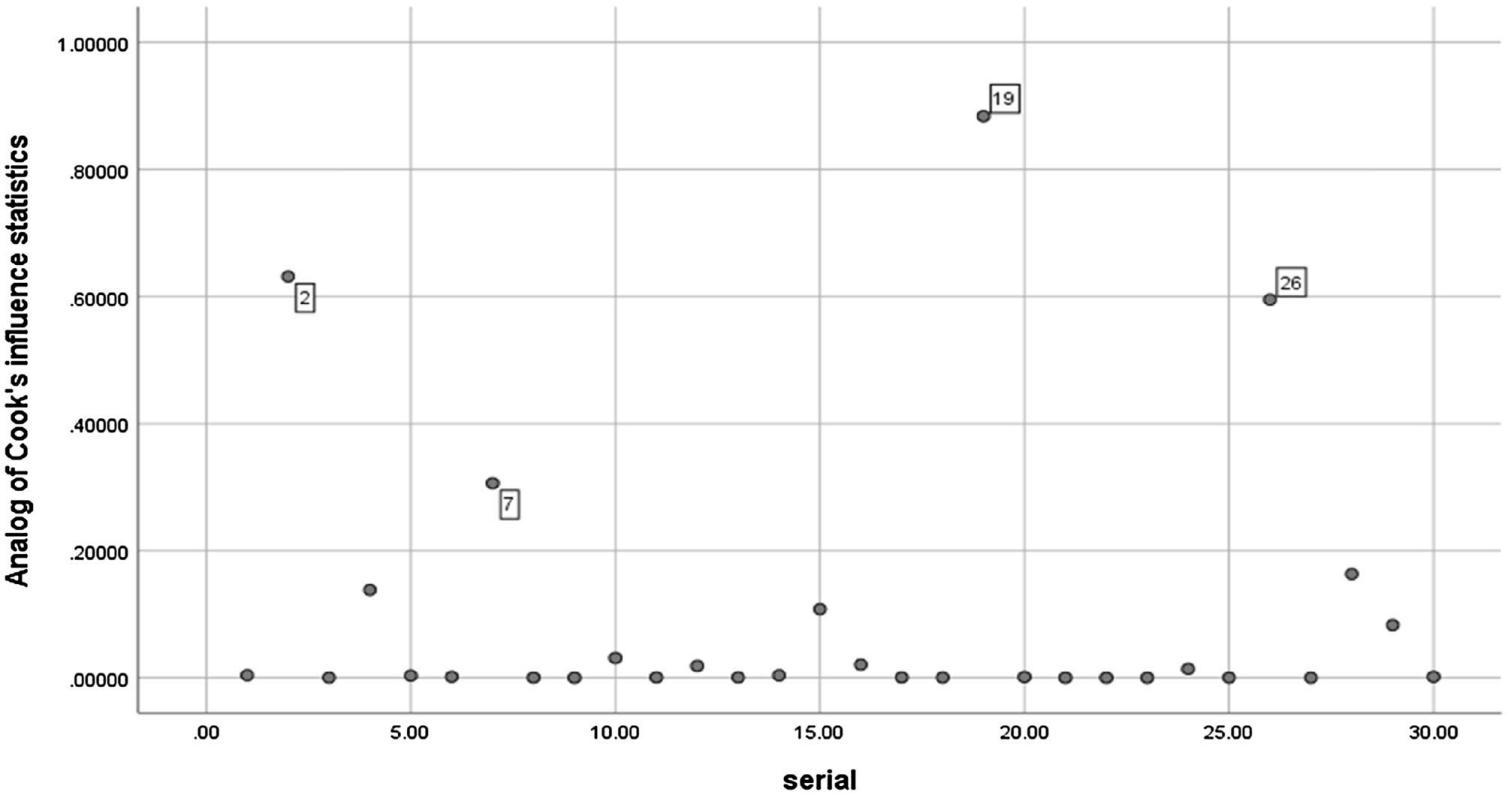
Scatterplot for influential outliers using Cook's test and serial cases.

Data were analyzed using IBM SPSS version 26, descriptive statistics in the form of mean, median, standard error, standard deviation, and percentages, Shapiro–Wilk test and Q–Q plots were applied to detect normality of variables, and Cook's distance test was plotted against serial number of cases to determine the influential outliers as shown in Fig. 3, cases number 2, 19, and 26 could affect our results and may be considered influential outliers and to confirm this effect, standardized DFFIT was applied and plotted against serial cases showing all cases were not deviated away from ± 3, so these values were not most probably influential. Bivariate correlations with Spearman rho and Pearson correlations were used.

Independent sample t-test and Mann Whitney U test were applied for the associations between continuous and two-groups categorical variables, while one way Manova test was applied for detecting differences of multiple continuous variables on dependent categorical variable (ISS stage) after applying homogeneity test to insure equality of variances (Levene's test), and equality of covariances (Box's test), in addition post hoc analysis was applied using Tukey test for pairwise comparison and Bonferroni correction was applied for pairwise comparison of estimated marginal means of PD1+CD8+T cells to adjust p-values between different ISS stages.

Multicollinearity diagnostics was done with VIF < 4, but the condition index > 10 for all immune cells and Microparticles due to the presence of autocorrelations between these variables, so to determine which one of these variables greatly affect the variation of response Logistic regression with forward LR method was applied changing ISS stage into dummy variables. Roc curve was also applied for different variables to find their cutoff points of achieving non CR, all data were considered significant at *p*-value < 5%.

## Results

Thirty patients with symptomatic MM and 19 healthy controls were enrolled in this study, with significant accumulations of TMPs (*p* < 0.0001), and CD138+MPs (*p* < 0.0001) in patients compared to controls, while no significant difference in PMPs (*p* = 0.07) among both groups, Table 1.

**Table 1:** Differential expressions of microparticles between patients and controls.

	Patients	Controls	*p*-value
TMPs	68.95 ± 1.4	54.86 ± 0.5	0.0001
PMPs	63.18 ± 2.1	56.79 ± 2.8	0.07
CD138+MPs	11.75 ± 0.4	4.75 ± 0.2	0.0001

TMPs: total microparticles; PMPs: platelet microparticles, data expressed as mean ± SE, independent sample t-test, and Mann Whitney U test for significance, *p* < 0.05.

Furthermore, patients with MM showed statistically significant higher percentages of CD8+T cells (*p* = 0.007), PD1+CD8+T cells (*p* < 0.0001), and PD1+CD4+T cells (*p* < 0.0001) compared to their controls, while healthy controls had a significantly higher CD4+T cells (*p* < 0.0001) than patients as shown in Table 2.

**Table 2:** Differential accumulation of T lymphocytes and PD 1 expressing T lymphocytes between study groups.

	Patients	Controls	*p*-value
CD8+T cells	21.2 ± 0.66	18.1 ± 0.9	0.007
CD4+T cells	43.1 ± 1.3	49.8 ± 0.5	0.0001
PD1+CD8+T cells	14.26 ± 0.5	7.5 ± 0.3	0.0001
PD1+CD4+T cells	12.7 ± 0.4	8.3 ± 0.6	0.0001

Data expressed as mean ± SE, independent sample t-test and Mann Whitney U test for significance, *p* < 0.05.

Among 30 patients with active symptomatic multiple myeloma, 7, 17, and 6 patients were in stages I, II, and III respectively, with 11 patients achieved complete response to Bortezomib based combinations while 19 patients did not achieve CR as illustrated in Table 3.

**Table 3:** Characteristics of 30 MM patients.

Factor	Mean ± SE
Hb (g/dL)	9.25 ± 0.3
Platelets	130.8 ± 9.2
LDH (U/L)	932.7 ± 65.6
Serum calcium (mg/dL)	11.7 ± 0.3
Total protein (g/L)	69.9 ± 1.0
Serum albumin (g/L)	30.5 ± 1.6
M protein (g/L)	43.5 ± 2.3
Urea (mg/dL)	45.1 ± 2.6
Creatinine (mg/dL)	1.9 ± 0.2
Bone marrow plasma	41.8 ± 3.0
Lytic bony lesions	≤ 1 = 8 patients
	> 1 = 22 patients
β2M (mg/mL)	4.4 ± 0.4
**ISS staging**	
Stage I	7 (23.3%)
Stage II	17 (56.7%)
Stage III	6 (20%)
**Response**	
CR	11/30
Non CR	19/30

LDH: lactate dehydrogenase; Hb: hemoglobin; MM: multiple myeloma; β2M: beta2 microglobulin; ISS: international staging system; CR: complete response.

Table 4 described different correlations between MPs, immune cells, and prognostic factors, with positive correlations were reported between PMP and PD1+CD4+T cells, CD138+MPs and CD8+T cells, PD1+CD8+T cells and PD1+CD4+T cells, PD1+CD8+T cells were positively correlated with LDH, M-protein, bone marrow plasma percentage, and β2 microglobulin, and β2 microglobulin was also correlated with PD1+CD4+T cells, furthermore PMP, CD138MPs, PD1+CD8+T cells, PD1+CD4+T cells, CD8+T cells, and CD4+T cells were positively associated with increased numbers of bony lesions.

**Table 4:** Correlations between MPs, PD1, and other prognostic factors of MM.

		TMPs	PMP	CD138+MP	PD1+CD8+	PD1+CD4+	CD8+	CD4+
TMPs	r*	NA	0.252	0.149	− 0.120	0.149	+ *0.547*	*− 0.466*
	p		0.179	0.432	0.526	0.432	*0.002*	*0.009*
PMP	r*	0.252	NA	0.295	0.248	+ *0.496*	0.254	0.123
	p	0.179		0.113	0.187	*0.005*	0.175	0.516
CD138+MP	r	0.149	0.295	NA	− 0.032	0.229	+ *0.514*	0.113
	p	0.432	0.113		0.868	0.224	*0.004*	0.551
PD1+CD8+	r	− 0.120	0.248	− 0.032	NA	+ *0.457*	0.112	0.332
	p	0.526	0.187	0.868		*0.011*	0.557	0.073
PD1+CD4+	r*	0.149	+ *0.496*	0.229	+ *0.457*	NA	0.306	− 0.026
	p	0.432	*0.005*	0.224	*0.011*		0.100	0.893
CD8	r	+ *0.547*	0.254	+ *0.514*	0.112	0.306	NA	− 0.215
	p	*0.002*	0.175	*0.004*	0.557	0.100		0.254
CD4	r*	*− 0.466*	0.123	0.113	0.332	− 0.026	− 0.215	NA
	p	*0.009*	0.516	0.551	0.073	0.893	0.254	
LDH	r	− 0.161	0.099	0.120	+ *0.609*	0.153	0.227	0.088
	p	0.395	0.602	0.527	< *0.001*	0.420	0.227	0.645
Serum calcium	r	0.165	0.282	0.027	0.319	0.183	0.150	0.049
	p	0.384	0.131	0.887	0.085	0.333	0.430	0.795
Serum albumin	r	− 0.198	− 0.252	− 0.204	− 0.296	− 0.190	− 0.191	0.214
	p	0.294	0.180	0.279	0.112	0.316	0.311	0.255
M protein	r	− 0.130	0.188	0.048	+ *0.703*	0.160	0.306	0.234
	p	0.494	0.320	0.801	< *0.001*	0.399	0.100	0.214
Creatinine	r	− 0.068	0.026	0.227	0.273	0.200	0.257	− 0.154
	p	0.721	0.890	0.228	0.144	0.288	0.170	0.416
Bone marrow plasma	r	− 0.150	0.158	0.314	+ *0.559*	0.353	0.097	0.114
	p	0.429	0.404	0.091	*0.001*	0.056	0.609	0.548
β2M	r	− 0.146	0.304	0.165	+ *0.839*	+ *0.450*	0.081	0.349
	p	0.441	0.102	0.384	< *0.001*	*0.013*	0.672	0.059
Total protein	r	0.353	− 0.111	− 0.157	− 0.057	0.242	0.255	*− 0.429*
	p	0.056	0.560	0.406	0.764	0.198	0.175	*0.018*
Bony lesions	r	0.259	+ *0.614*	+ *0.421*	+ *0.620*	+ *0.581*	+ *0.380*	0.190
	p	0.2	< *0.001*	*0.02*	< *0.001*	*0.001*	*0.037*	0.3

r: Pearson correlation coefficient; r*: Spearman rho correlation coefficient; p: p-value; MP: microparticles; β2M: beta2 microglobulin.

**Figure 4:**
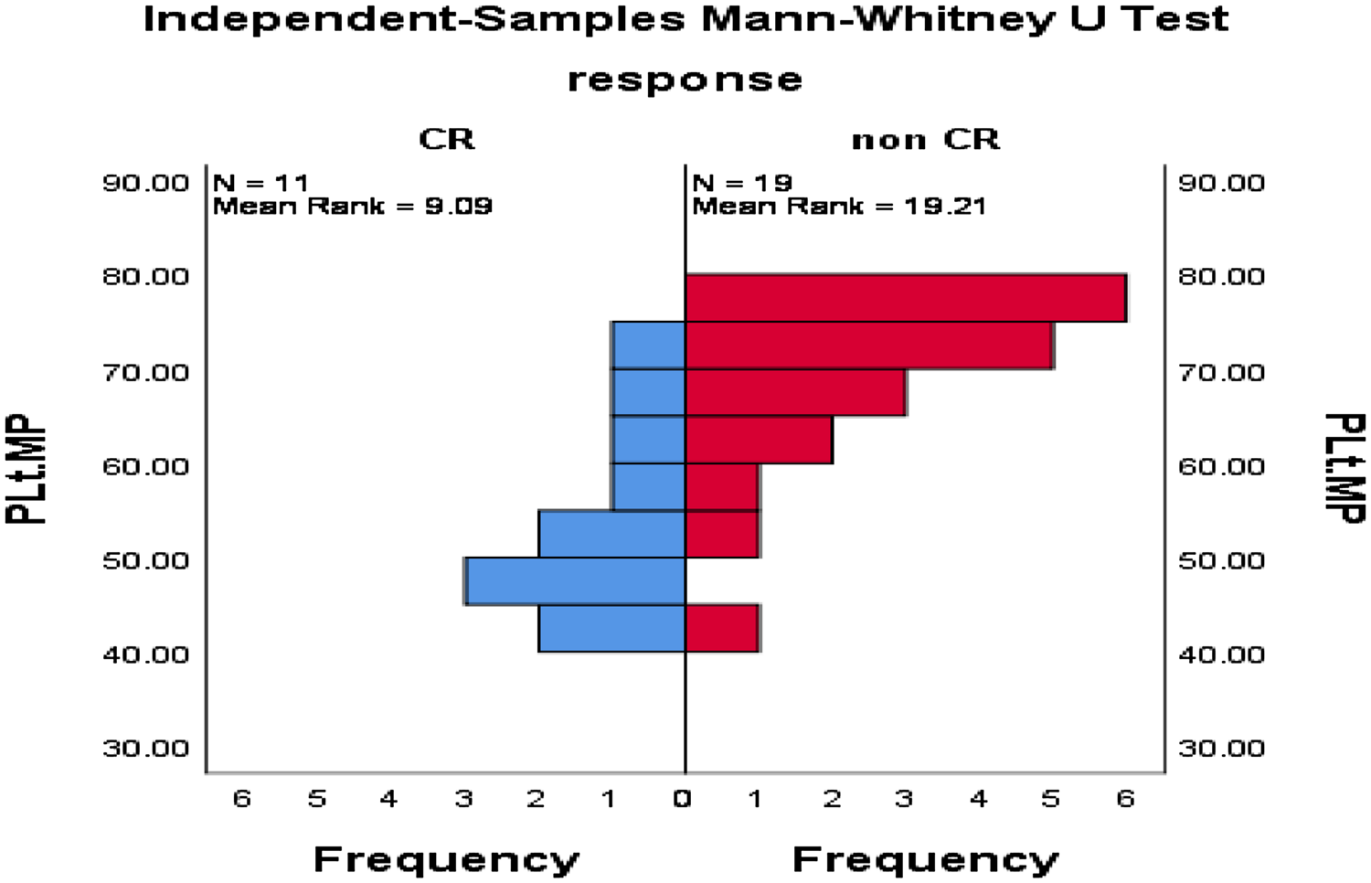
Significant difference in PMP among MM patients with CR versus those without CR, *p* = 0.002.

**Figure 5:**
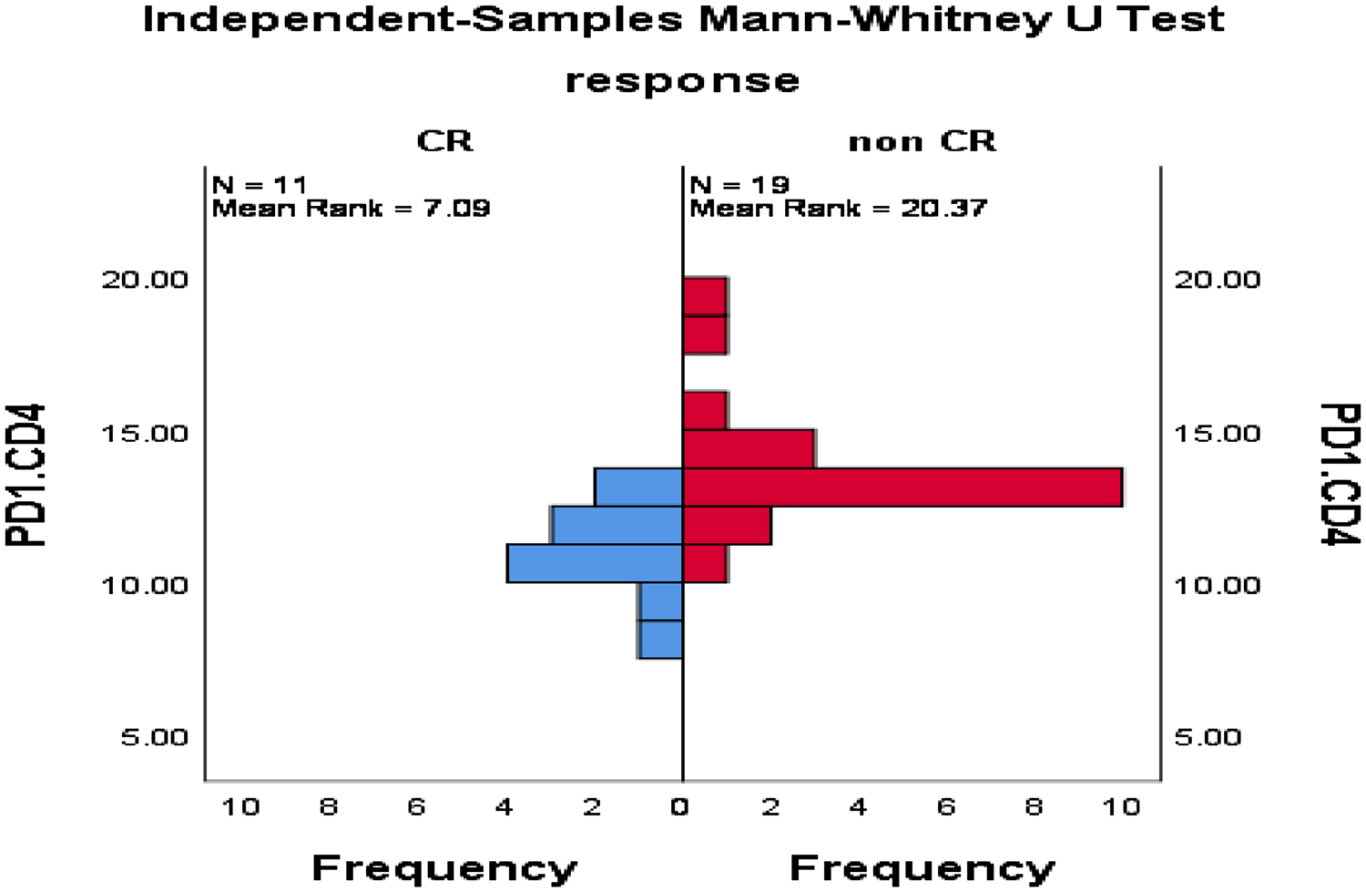
Significant difference between MM patients with CR and those without CR regarding PD1+CD4+T cells, *P* < 0.0001.

**Figure 6:**
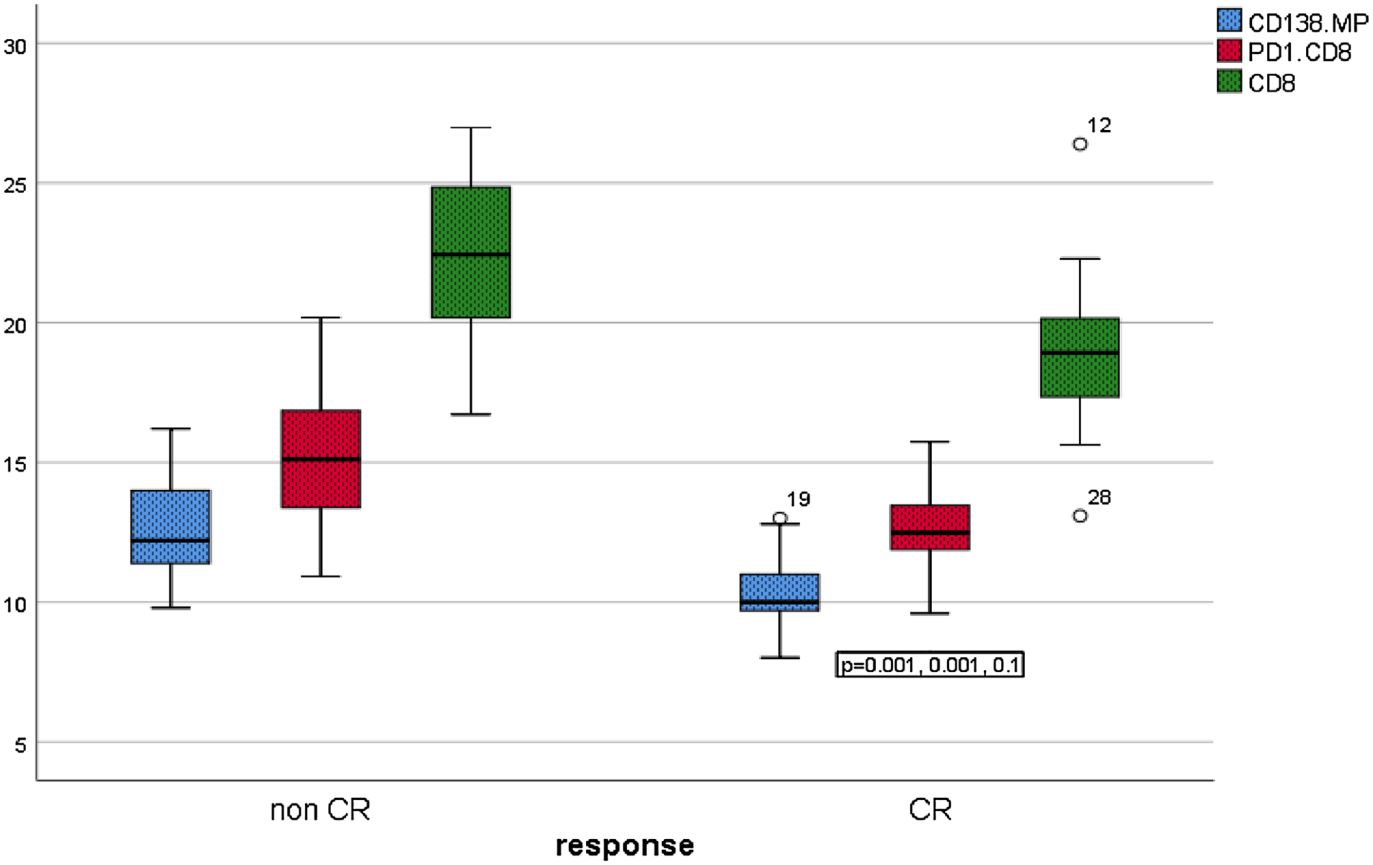
Boxplots showed significant difference between response groups of MM patients regarding CD138MP, PD1+CD8+T, and CD8+T cells.

As shown in Table 5, and Figs. 4, 5, 6, patients with CR had significantly lower PMP, CD138+MP, PD1+CD8+T cells, PD1+CD4+T cells, and CD8+T cells compared with patients without CR, while no significant difference in TMP and CD4+T cells between response groups.

**Table 5:** Impact of MPs and PD1 expressing lymphocytes on the response of 30 patients with active MM.

	Response	Mean ± SE	*p*-value
TMPs	CR	67.6 ± 2.5	0.61
	Non CR	69.7 ± 1.6	
PMP	CR	54.4 ± 3.1	0.002
	Non CR	68.3 ± 2.1	
CD138.MP	CR	10.3 ± 0.5	0.001
	Non CR	12.7 ± 0.4	
PD1+CD8+T	CR	12.7 ± 0.5	0.01
	Non CR	15.2 ± 0.6	
PD1+CD4+T	CR	10.9 ± 0.4	< 0.0001
	Non CR	13.7 ± 0.5	
CD8+T	CR	19.0 ± 1.1	0.01
	Non CR	22.4 ± 0.7	
CD4+T	CR	42.1 ± 2.2	0.52
	Non CR	43.7 ± 1.6	

TMP: total microparticles; PMP: platelet microparticles; CR: complete response; PD1: programmed death-1; Mann Whitney test was applied for TMP, PMP, PD1+CD4+T, and CD4 and independent sample t-test was applied for the remaining variables.

**Figure 7:**
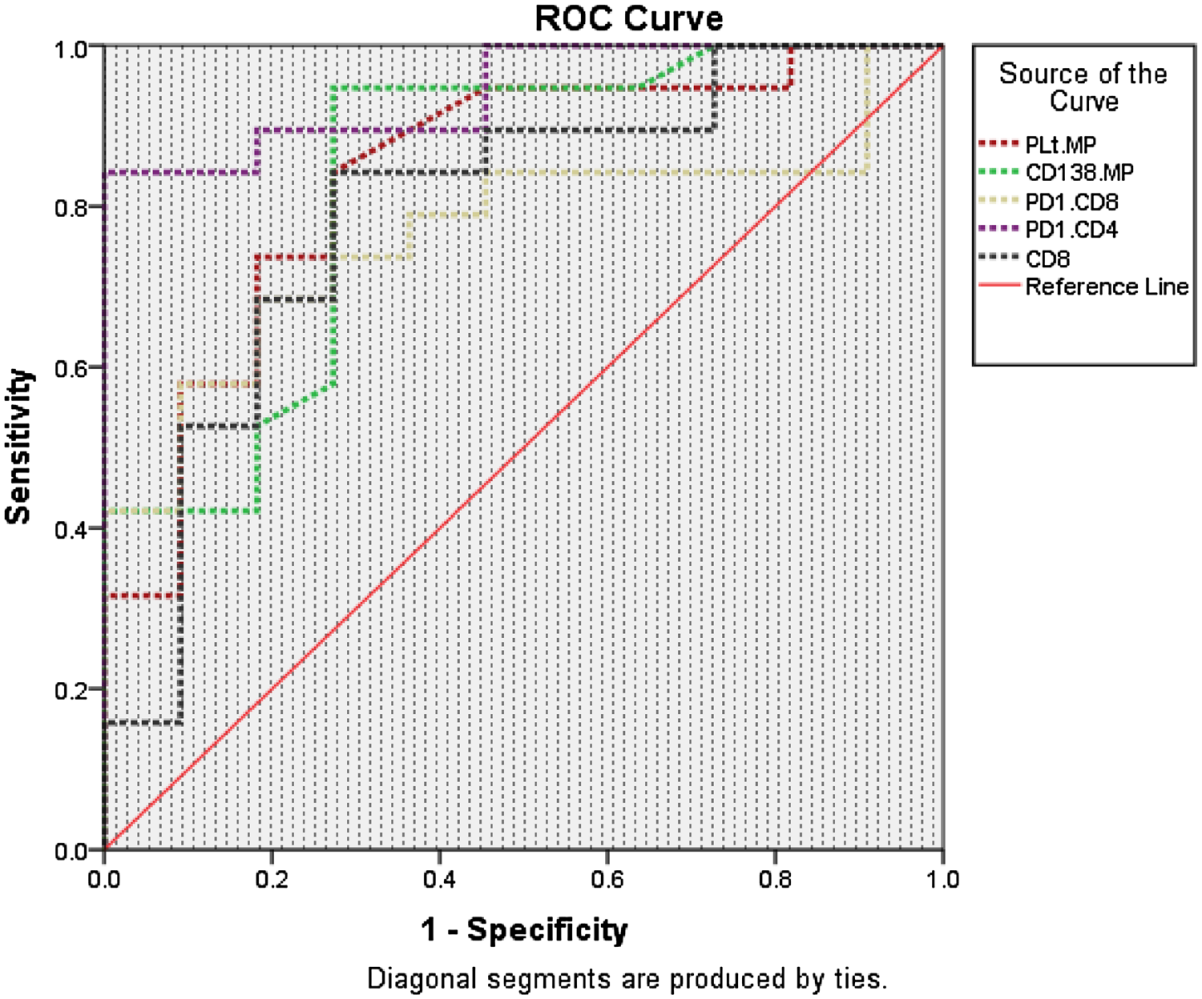
Receiver operating characteristics (ROC) curve comparing the performance of different MPs and immune cells in the prediction of the response of MM patients.

Receiver Operating Characteristic (ROC) curve analysis has shown that among different MPs and immune cells, PMP, CD138+MP, PD1+CD8+T cells, PD1+CD4+T cells, and CD8+T have shown good accuracy in predicting the response to Bortezomib based combination in patients with MM as illustrated in Table 6, and Fig. 7, the largest AUC was observed with PD1+CD4+T cells for predicting non CR among active myeloma patients (AUC = 0.94, *p* = 0.0001) with the highest sensitivity (92%), specificity (80%), and performance (72%).

**Table 6:** Performance of different MPs and immune cells in MM.

Variable	AUC	Cut off	Sensitivity	Specificity	Youden index	*p*-value
PMP	0.84	≥ 61	83	74	57	0.002
CD138+MP	0.83	≥ 10.6	91	74	65	0.003
PD1+CD8+T cells	0.77	≥ 13.5	71	75	46	0.017
PD1+CD4+T cells	0.94	≥ 11.3	92	80	72	0.0001
CD8+T cells	0.79	≥ 20.1	83	70	53	0.008

AUC: area under the curve; PMP: platelet microparticles; MP: microparticles; PD1: programmed death-1.

### Differences of MPs and immune cells across ISS stages

**Figure 8:**
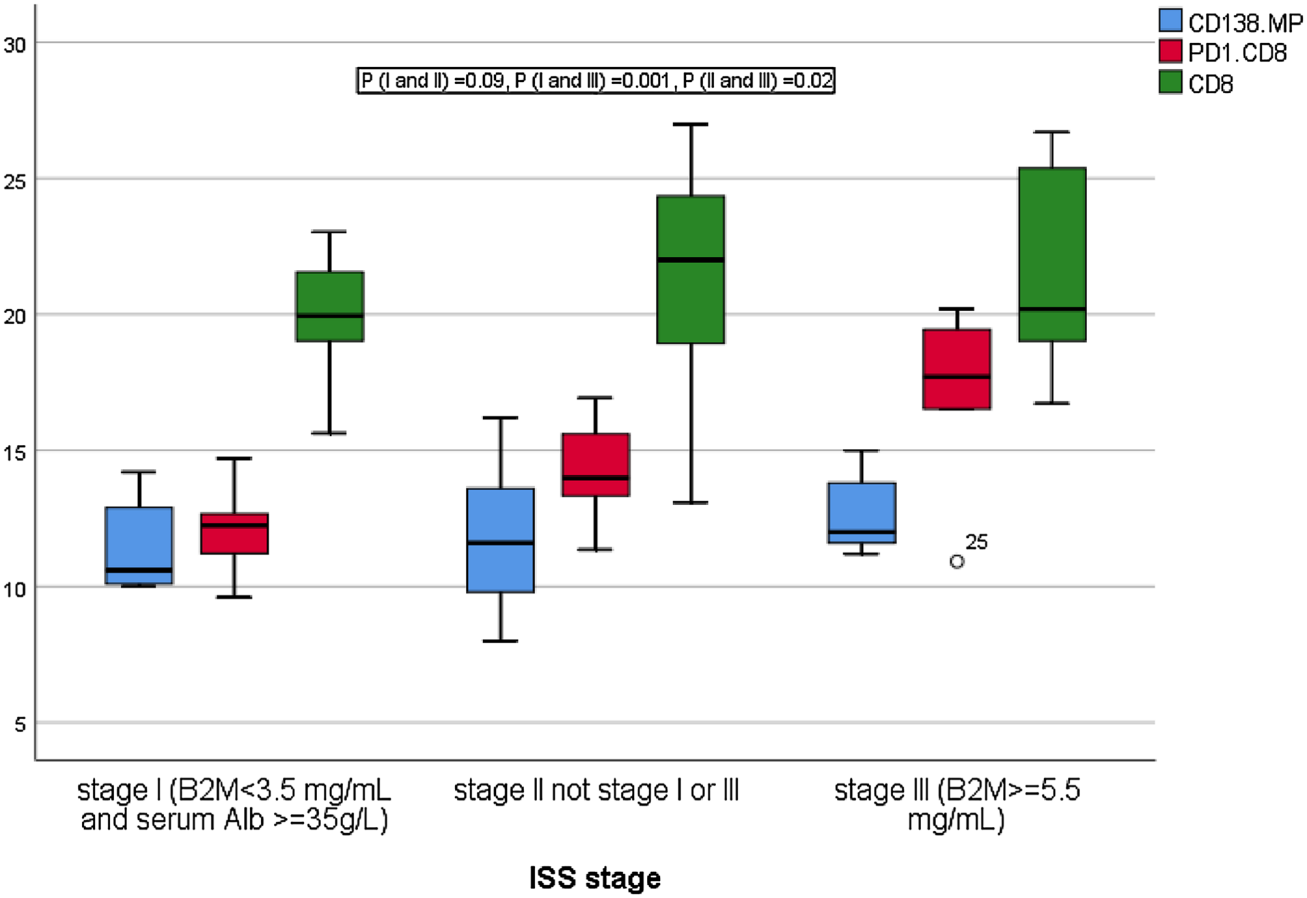
Tukey HSD for post hoc analysis was applied for PD1+CD8+T cells to detect differences across different ISS stages; significant accumulation of PD1+CD8+T cells was found in stage III compared to stage I (mean difference = 5.04, *p* = 0.001), and stage II (mean difference = 2.9, *p* = 0.018), while no significant difference between stage I and II (mean difference = 2.1, *p* = 0.07), Bonferroni correction was applied to adjust p-values to be 0.09, 0.020, and 0.001 for differences of estimated marginal means between stages I and II, II and III, and III and I respectively.

One way Manova with Tukey test for post hoc analysis was applied after performing Levene's test of homogeneity to assess equality of variance for CD138+MP, CD8, PD1+CD8+T cells (*p* = 0.3, 0.4, 0.2), and Box's test to assess equality of dependent covariance across all stages (*p* = 0.316), generally there was a statistically significant effect of all three variables on ISS stage (*F*(6,50) = 3.251, *p* = 0.009, Wilks' Lambda = 0.517, *p*-value = 0.009, partial η^2^ = 0.281); no significant differences were detected for CD138+MP (*p* = 0.6) and CD8 (*p* = 0.5), there were significant effect of ISS staging on PD1+CD8+T cells (*F*(2, 27) = 9.467, *p* = 0.001, partial η^2^ = 0.412), Table 7, Fig. 8.

**Table 7:** Multivariate analysis of normally distributed variables on ISS stage.

Factor	Dependent	F-statistics	df	R^2^	*p*-value
ISS stage	CD138MP	0.428	2	0.34	0.6
	PD1+CD8+T	9.467	2	0.412	0.001
	CD8+T cells	0.524	2	0.037	0.5

df: degrees of freedom; ISS: international staging system; PD1: programmed death-1; one way Manova for significance, *p* < 0.05.

**Figure 9:**
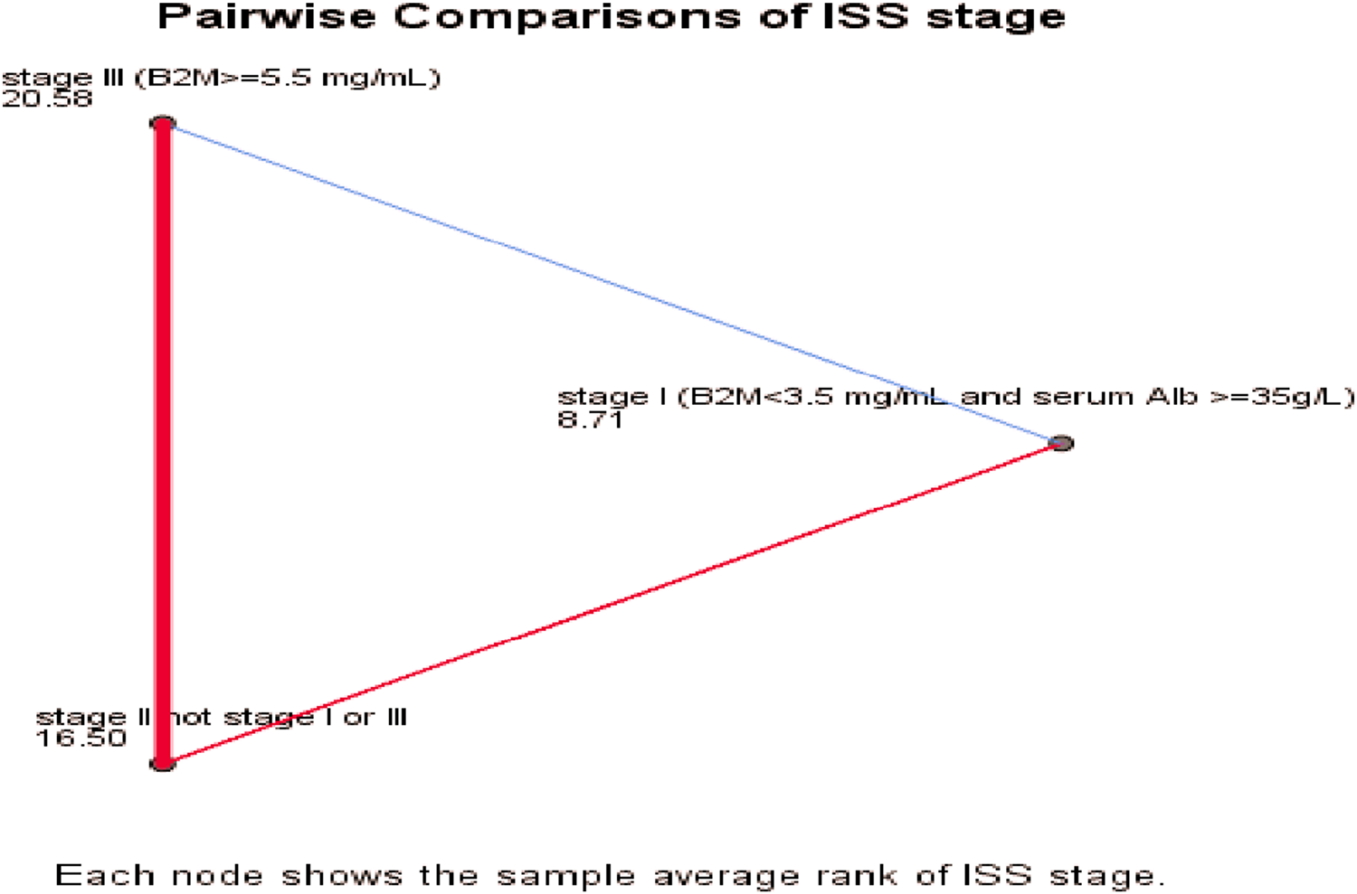
Significant accumulation of PD1+CD4+T cells in stages II and III compared to stage I (*p* = 0.049, *p* = 0.015 respectively), while no significant differences between stages II and III (*p* = 0.3).

Independent sample Kruskal Wallis test was applied for other Microparticles and immune cells; no significant differences of the distributions of TMP, PMP, and CD4+T cells across different stages of MM patients (*p* = 0.8, 0.6, 0.07 respectively), however significant difference in the distribution of PD1+CD4+T across ISS stages was detected (*p* = 0.041) as shown in Fig. 9.

### Logistic regression

Due to autocorrelations between MPs and immune cells, logistic regression with forward LR method was carried out, the overall prediction rate of the model for response to treatment was 90% as shown in Table 8, for each point of increase in PD1+CD4+T cells there was a decrease in the odds of CR by 15.5%, *p* = 0.025, also for each point of increase in CD138+MP there was a decrease in the odds of CR by 47.2%, *p* = 0.059, Table 9, furthermore other variables were removed from the model because of autocorrelation and no added value in the prediction rate of model.

**Table 8:** Model summary of logistic regression done for 30 patients with MM.

Independent variables	Dependent variables	R^2^	Chi^2^	*p*-value	df	% of correct classification
TMP	Response	Step 1 = 0.653	19.487	< 0.0001	1	Step 1 = 83.3%
PMP						
CD138MP						
PD1CD8		Step 2 = 0.77	5.509	= 0.019	1	Step 2 = 90%
PD1CD4						
CD4						
CD8						

Response is a binary variable with two categories; 0; non CR, 1; CR, df; degrees of freedom, *p* < 0.05.

**Table 9:** Variables in the Equation of logistic regression (Ln (dependent = CR) = 30.621 – 0.751 * 1 – 1.864 * 2).

Variables		B	S.E.	Wald	df	*p*-value	Exp (B)	95% C.I. for Exp(B)	
								Lower	Upper
Step 1^a^	PD1+CD4+T	− 1.790	0.654	7.500	1	*0.006*	0.167	0.046	0.601
	Constant	21.361	7.997	7.135	1	0.008	1,891,728,042.290		
Step 2^b^	CD138+MP	− 0.751	0.398	3.553	1	0.059	0.472	0.216	1.030
	PD1+CD4+T	− 1.864	0.834	5.001	1	*0.025*	0.155	0.030	0.794
	Constant	30.621	12.083	6.423	1	0.011	19,892,982,541,237.910		

^a^Variable(s) entered on step 1: PD1+CD4+T cells, ^b^Variable(s) entered on step 2: CD138+MP, Exp(B); odds ratio, CI; confidence interval, df; degrees of freedom, *p* < 0.05.

## Discussion

Multiple myeloma is a genetically heterogeneous clonal plasma cell disease which is substantially preceded by an asymptomatic premalignant stage, monoclonal gammopathy of undetermined significance (MGUS), to clinically aggressive stage with overt clinical pictures^17^. Among all cancers, it represents about 1%, in spite, a minority of them achieved sustained complete response for a prolonged period or the so-called operational cure^18^, even if they achieve this cure they will continue on suppressive therapy to fight against the risk of relapse with no clear plateau in overall survival^19^.

Our results elucidated significant higher levels of TMP, CD138+MP, T cell subsets, PD1 expressed on T cells in active MM patients compared with controls, also there were significant positive correlations between PMP and PD1+CD4+T cells, CD138+MP and CD8, and PD1+CD8+T cells and all of the followings LDH, M protein, BM plasma level, and β2 microglobulin, furthermore, there were significantly elevated levels of PMP, CD138MP, PD1, CD8+T cells in patients who did not achieve CR with specific cutoff values as mentioned in Table 6.

Immune checkpoint blockade is proposed to be effective in many cancers where immune deregulation due to increased expression of negative co-stimulation of cells in tumor microenvironment plays important role in tumor progression and resistance to treatment, multiple myeloma is an example of these cancers with progressive immune dysregulation characterized by loss of myeloma reactive T-cell population, decreased antigen presenting and effector cell functions, and BM microenvironment that promotes immune escape^20,21^. Preclinical data have confirmed the important role of the PD-1 pathway in immune evasion by MM cells^22^, anti-PD1 and anti-PD-L1 monoclonal antibodies exhibited objective responses and antitumor activity in relapsed and refractory MM in phase I studies^23,24^.

Batorov et al. reported significantly higher levels of PD1+CD4+T cells, PD1+CD8+T cells, CD4+T cells, and CD8+T cells in multiple myeloma patients compared to healthy donors^25^, On the contrary, Sponaas et al. did not detect any difference of PD1 expressed on CD8+T cells between myeloma patients and healthy controls, furthermore, high expression of PD1+CD8+T cells was not found to correlate with tumor load to suggest that these cells were specific for non-myeloma antigens^26^, our results came in alignment with Batorov et al. with exception of CD4+ which was higher in healthy controls compared to patients.

Physiologically, MPs take part in cell signaling and swapping of proteins and nucleic acids between cells, also are involved in the intercellular crosstalk, elevated levels of MPs are detected in many pathological conditions including inflammation, vascular diseases, diabetes, and cancers where they act as a surrogate marker for disease activity especially in poorly accessible tissues^27–30^, it is well documented that immunomodulatory drugs play important role in treatment of MM to be involved in many treatment regimens, however the risk of thromboembolism increased with their use to focus on the clinical significance of MP especially PMP^29^.

Studies reported that TMP, PMP, and CD138+MP were elevated in patients with MM compared to healthy controls with significant prognostic potential of CD138+MPs in predicting the response to treatment and risk of relapse^31^, our results agreed with the previous study.

Our results showed no significant differences in the total MP among response groups to demonstrate that they may predict the disease state of MM patients compared to healthy controls rather than response state.

MPs emerged as a surrogate marker for response in many cancers including MM^32,33^, our results failed to consider MPs as a marker for disease progression because of absence of significant differences of MPs across different ISS stages, in spite there were significant association between PMPs and CD138 MPs with increased bony lesions, this study adds to this body of research and provides support for the use of MPs as a novel prognostic for response assessment in MM.

Crosstalk between tumor cells and immune cells in tumor microenvironment is critical for tumor progression; in addition, tumor cells are capable to hijack immune cells especially innate immunity, subsequently MPs released from tumor cells, not only contain messenger molecules, enzymes, RNAs, and even DNA, but also are capable of transferring these bioactive molecules from one cell to another, subsequently act as a vector for transferring messages between tumor cells and possibly immune cells to acquire aggressive phenotype^34,35^. This crosstalk rather appeared in the current study where significant positive correlations between TMP and CD8+T cells, PMP and PD1+CD4+T cells, and CD138+MP and CD8+T cells while negative correlation between TMP and CD8+T cells.

In solid tumors, MPs were accused to increase chemotherapy resistance by inducing inflammation, metastasis, and angiogenesis^36^. Furthermore, in response to chemotherapy, these MPs act as a getaway for BM-derived cells to be mobilized and homed to metastatic sites^37^, recently, supporting evidences to our opinion in the contributing role of MPs to chemoresistance came from the findings of their expression of PD-L1 with subsequent immune evasion from T-cell inhibition and T-cell overexpression of PD-1 especially after treatment^38,39^.

In one study, MPs from irradiated breast carcinoma could express high levels of immunosuppressive proteins compared with unirradiated cells and modulate immune evasion partially by expression of PD-L1 with enhanced tumor growth; the study suggested that PD-L1 and MPs could be a possible biomarker to identify breast cancer patients who were likely benefited from radiotherapy and immunotherapy^40^.

To our knowledge, our study was the first one to address the relations between Microparticles and immune cells in MM, and the added impact of these relations in resistance to treatment and disease progression, further studies with thorough methodology and adequate sample size may augment these relations.

## Conclusion

We have demonstrated elevated levels of MPs and immune cells in MM patients compared to healthy controls, also the levels of these cells and particles were lower in patients achieved CR compared to those without CR, Microparticles and PD1 expressions were associated with proliferative potential and resistance to Bortezomib-based treatments, our results suggested that they played a crucial role in myeloma progression, in addition this study provides support for the potential prognostic value of MPs and the possible interplay between MPs and PD1 in multiple myeloma.

## Data Availability

All data generated or analyzed during this study are included in this submitted article.
